# Combined ethanolic extracts of *Camellia sinensis* and *Curcuma longa* exhibit ovicidal and anthelmintic activities against *Fasciola gigantica* under *in vitro* conditions

**DOI:** 10.14202/vetworld.2026.2463-2478

**Published:** 2026-06-20

**Authors:** Sefi Lestyo Harini, Eka Pramyrtha Hestianah, Lucia Tri Suwanti, Mufasirin Mufasirin, Kadek Rachmawati, Diky Ramdani, Anuraga Jayanegara, Aswin Rafif Khairullah, Nanik Hidayatik

**Affiliations:** 1 Master Program of Veterinary Disease and Public Health, Faculty of Veterinary Medicine, Universitas Airlangga, Jl. Dr. Ir. H. Soekarno, Kampus C Mulyorejo, Surabaya, 60115, East Java, Indonesia; 2 East Java Animal, Fish and Plant Quarantine, Indonesia Quarantine Agency, Jl. Raya Bandara Ir. H Juanda no. 26, Semawalang, Semambung, Gedangan, Sidoarjo, 61253, East Java, Indonesia; 3 Division of Veterinary Anatomy, Faculty of Veterinary Medicine, Universitas Airlangga, Jl. Dr. Ir. H. Soekarno, Kampus C Mulyorejo, Surabaya, 60115, East Java, Indonesia; 4 Department of Veterinary Parasitology, Faculty of Veterinary Medicine, Universitas Airlangga, Jl. Dr. Ir. H. Soekarno, Kampus C Mulyorejo, Surabaya, 60115, East Java, Indonesia; 5 Department of Basic Veterinary Medicine, Faculty of Veterinary Medicine, Universitas Airlangga, Jl. Dr. Ir. H. Soekarno, Kampus C Mulyorejo, Surabaya, 60115, East Java, Indonesia; 6 Department of Animal Production, Faculty of Animal Husbandry, Universitas Padjadjaran, Jl. Raya Bandung - Sumedang KM 21, Sayang, Jatinangor, Sumedang, 45363, West Java, Indonesia; 7 Department of Animal Nutrition and Feed Technology, Faculty of Animal Husbandry, IPB University, Jl. Raya Darmaga Kampus IPB, Babakan, Dramaga, Bogor, 16680, West Java, Indonesia; 8 Research Center for Veterinary Science, National Research and Innovation Agency (BRIN), Jl. Raya Bogor Km. 46 Cibinong, Bogor, 16911, West Java, Indonesia

**Keywords:** anthelmintic activity, *Camellia sinensis*, *Curcuma longa*, *Fasciola gigantica*, fasciolosis, medicinal plants, ovicidal activity, phytochemicals

## Abstract

**Background and Aim:**

Fasciolosis caused by Fasciola gigantica remains a major parasitic disease affecting ruminant livestock, leading to substantial economic losses through decreased productivity, liver condemnation, and mortality, while also posing a zoonotic threat. The prolonged and indiscriminate use of synthetic anthelmintics has contributed to the emergence of drug-resistant parasite populations, emphasizing the urgent need for alternative and sustainable control strategies. Medicinal plants rich in bioactive phytochemicals have gained attention as potential natural anthelmintic agents. Green tea (Camellia sinensis) contains tannins and catechins with antiparasitic properties, whereas turmeric (Curcuma longa) is rich in curcuminoids known to disrupt parasite metabolism and structural integrity. Therefore, this study aimed to evaluate the combined ovicidal and anthelmintic activities of ethanolic extracts of green tea and turmeric against F. gigantica under in vitro conditions.

**Materials and Methods:**

Adult worms and eggs of *F. gigantica* were collected from naturally infected cattle. Six treatment groups were established, including physiological saline as the negative control, nitroxynil 10% as the positive control, and four combinations of green tea and turmeric extracts at different concentrations. Ovicidal activity was evaluated based on egg degeneration after 16 days of incubation, whereas adulticidal activity was assessed through worm mortality at multiple exposure times. Histopathological examination using hematoxylin and eosin staining and ultrastructural evaluation using scanning electron microscopy were performed to identify tissue and tegumental alterations. Statistical analysis was conducted using one-way analysis of variance followed by Duncan’s multiple range test.

**Results:**

The combined extracts demonstrated concentration- and time-dependent ovicidal and anthelmintic activities against *F. gigantica*. The highest extract concentration produced the greatest percentage of egg degeneration, characterized by eggshell rupture, membrane damage, abnormal morphology, and failure of embryonic development. Adult worm mortality increased progressively with exposure time, and complete mortality was observed at prolonged exposure periods in all extract-treated groups. Histopathological and ultrastructural analyses revealed marked tegumental disruption, spine deformation, erosion of the oral and ventral suckers, rupture of the intestinal lumen, and surface exfoliation in treated worms. Although nitroxynil induced more rapid effects, the combined extracts exhibited substantial antiparasitic activity at higher concentrations and longer exposure times.

**Conclusion:**

Combined ethanolic extracts of green tea and turmeric exhibited significant ovicidal and anthelmintic activities against *F. gigantica* under *in vitro* conditions. The findings suggest that these plant-derived extracts may serve as promising natural alternatives for controlling fasciolosis and warrant further phytochemical characterization and *in vivo* evaluation to confirm their efficacy, safety, and practical applicability in livestock systems.

## INTRODUCTION

Helminth infections remain a major global constraint to animal health and productivity, particularly in ruminant livestock systems [1]. These infections cause substantial socioeconomic losses through reduced weight gain, decreased milk and meat production, impaired reproductive performance, increased susceptibility to secondary infections, and mortality [2]. Both clinical and subclinical helminthiasis contribute significantly to production inefficiencies, leading to increased treatment costs and long-term economic burdens on farmers, especially in developing countries [3].

Fasciolosis is one of the most important helminth diseases affecting livestock worldwide and is caused by the liver flukes *Fasciola hepatica* and *Fasciola gigantica* [4]. Among these species, *F. gigantica* predominates in tropical and subtropical regions, including Southeast Asia [5]. The disease is widely distributed and has been reported with considerable prevalence in cattle populations, resulting in severe economic losses due to liver condemnation, reduced productivity, and animal mortality [6]. Globally, the economic impact of fasciolosis is estimated to reach billions of US dollars annually [7]. In addition to its veterinary importance, fasciolosis is recognized as a neglected zoonotic disease, with millions of human infections reported worldwide [8]. Although human cases have been documented in several Southeast Asian countries, comprehensive reports from Indonesia remain limited, highlighting the need for further attention to this parasitic disease [9].

Control of fasciolosis relies predominantly on the use of synthetic anthelmintic drugs, particularly triclabendazole, which is considered highly effective against both immature and adult stages of *Fasciola* spp. [10]. However, prolonged and indiscriminate use of anthelmintics has led to the emergence of drug resistance, raising serious concerns regarding the sustainability of current control strategies [11]. Anthelmintic resistance has been increasingly reported in *Fasciola* populations, reducing treatment efficacy and emphasizing the urgent need for alternative, environmentally friendly, and sustainable control approaches [12].

Medicinal plants have gained increasing attention as potential sources of natural anthelmintic agents because of their bioactive secondary metabolites, lower environmental impact, and reduced risk of resistance development [13]. Several plant-derived compounds, including tannin-rich, alkaloid-containing, and essential oil-based extracts, have demonstrated variable success against trematodes and nematodes, although efficacy often depends on plant species, extraction method, and parasite developmental stage. Despite these promising findings, many previous studies have been limited by inconsistent efficacy, lack of mechanistic evaluation, insufficient standardization of extract combinations, and inadequate validation against multiple parasite life stages. Moreover, most studies have focused either on egg stages or adult worms alone, resulting in a limited understanding of the broader antiparasitic potential of combined phytotherapeutic formulations.

Green tea (*Camellia sinensis*) is rich in polyphenolic compounds, particularly tannins and catechins, which have demonstrated antiparasitic and anthelmintic activities in several studies [14–16]. Tannins are known to interfere with parasite metabolism, disrupt tegument integrity, and impair egg viability, making them promising candidates for helminth control [17]. Previous studies have reported the effectiveness of green tea extracts against nematodes and other parasitic worms [18]. Similarly, turmeric (*Curcuma longa*) is widely used in traditional medicine and contains curcumin and various terpenoids as its major bioactive compounds [19]. Curcumin has been reported to exert antiparasitic effects by inhibiting parasite motility, inducing structural damage, and disrupting essential physiological processes [20]. Several studies have demonstrated the anthelmintic activity of turmeric extracts against trematodes and other helminths, including *Fasciola* spp., as well as nematode larvae [21–23].

Recent evidence suggests that combining different plant extracts may produce additive or potentially synergistic effects, thereby enhancing overall anthelmintic efficacy through complementary mechanisms of action [24]. The interaction among multiple phytochemicals may improve bioactivity compared with single-extract treatments [25]. In the present context, the combination of tannin-rich green tea and curcuminoid-containing turmeric was selected for their potentially complementary mechanisms, including tegumental disruption, interference with parasite metabolism, induction of oxidative stress, and impairment of egg integrity.

Although combinations of plant-based extracts have shown promising results against several helminth species, substantial research gaps remain regarding their application against *F. gigantica*. Previous studies primarily evaluated individual plant extracts, focused on gastrointestinal nematodes, or assessed only one developmental stage of the parasite. Furthermore, available studies rarely integrated ovicidal evaluation, adulticidal assessment, histopathological examination, and ultrastructural analysis within a single experimental framework. Importantly, previous work by Hidayatik *et al*. [26] investigated similar herbal extracts in sheep-associated gastrointestinal parasites, which differ biologically, anatomically, and physiologically from liver flukes. Therefore, the efficacy of these combined extracts against trematodes such as *F. gigantica* cannot be directly extrapolated from earlier findings.

In addition, limited information is available regarding the structural and ultrastructural alterations induced by combined herbal extracts in *F. gigantica*. Most previous studies relied primarily on mortality observations without comprehensive histological or scanning electron microscopy (SEM)-based confirmation of tegumental and internal tissue damage. The absence of integrated evaluations has restricted mechanistic understanding of how combined phytochemicals affect parasite survival and egg development. Moreover, the comparative efficacy of combined green tea and turmeric extracts against both egg and adult stages of *F. gigantica* under *in vitro* conditions remains poorly documented. To the best of our knowledge, no previous study has specifically evaluated the combined ethanolic extracts of green tea and turmeric against both ovicidal and adulticidal stages of *F. gigantica* using integrated mortality, histopathological, and SEM assessments.

Therefore, this study aimed to evaluate the ovicidal and anthelmintic activities of combined ethanolic extracts of green tea and turmeric against *F. gigantica* under *in vitro* conditions. Specifically, this study investigated the effects of different extract combinations on egg degeneration, inhibition of embryonic development, and adult fluke mortality over time. In addition, histopathological examination and SEM analyses were performed to characterize structural and ultrastructural alterations induced by the treatments.

By targeting both egg and adult stages of *F. gigantica*, this study sought to provide broader evidence regarding the potential application of combined plant-based extracts as alternative anthelmintic agents for fasciolosis control. The findings are expected to contribute to the development of environmentally friendly and sustainable antiparasitic strategies, while also establishing a scientific basis for future phytochemical characterization, mechanistic investigations, dosage optimization, and *in vivo* validation of combined herbal formulations against fasciolosis.

## MATERIALS AND METHODS

### Ethical approval

Ethical approval was not required for this study because all parasite materials were obtained from abattoir-derived cattle organs collected during routine slaughterhouse inspections, and no live animals were directly handled, restrained, or experimentally manipulated during the study. The samples used in this investigation consisted exclusively of post-mortem biological materials collected from cattle slaughtered for commercial purposes at a municipal abattoir. Therefore, the study did not involve experimental animal procedures or interventions requiring ethical clearance under institutional animal welfare regulations.

In addition, parasite specimen collection was conducted in accordance with standard biosafety and laboratory handling procedures to ensure the safe processing of biological materials. The use of post-mortem samples was considered exempt from formal animal ethics committee review, in accordance with institutional guidelines and national recommendations regarding the use of slaughterhouse-derived specimens for laboratory-based parasitological research.

### Study period and location

The study was conducted from July to December 2024. Adult worms and eggs of *F. gigantica* were collected from naturally infected cattle slaughtered at the municipal abattoir of Ende Regency, East Nusa Tenggara, Indonesia. Samples were obtained from multiple naturally infected cattle of the same species (*Bos taurus*) during routine post-mortem inspection to minimize host-specific sampling bias. *In vitro* ovicidal and anthelmintic assays were conducted at the Animal Quarantine Laboratory, Agricultural Quarantine Agency of East Nusa Tenggara.

Preparation of green tea leaf powder and turmeric rhizome powder was carried out at the Faculty of Animal Science, Universitas Padjadjaran, Indonesia. Extraction of plant materials was performed at the Pharmacology Laboratory, Faculty of Veterinary Medicine, Universitas Airlangga, Indonesia. Histological slide preparation was conducted at the National Veterinary Standard Testing Center, Bogor, Indonesia, whereas histological examination was carried out at the Animal Quarantine Laboratory, East Nusa Tenggara, Indonesia. SEM analysis was performed at the Integrated Science Area Laboratory, National Research and Innovation Agency, Cibinong, Indonesia, and at the Integrated Research and Testing Laboratory, Universitas Gadjah Mada, Indonesia. The study was conducted from July to December 2024.

### Study design

This study was conducted as an *in vitro* laboratory-based experimental study to evaluate the ovicidal and anthelmintic activities of combined ethanolic extracts of green tea and turmeric against eggs and adult worms of *F. gigantica*. The experiment employed a completely randomized design with six treatment groups, including negative and positive controls, and four replicates per treatment. The experimental design was established to assess concentration-dependent effects of the combined extracts on egg degeneration, adult worm mortality, histopathological alterations, and ultrastructural damage.

### Parasite collection

Adult *F. gigantica* worms were recovered from the livers of naturally infected cattle during post-mortem inspection. Morphological identification was based on the typical elongated leaf-like body shape, prominent cephalic cone, broad shoulders, and body proportions consistent with standard taxonomic descriptions of *F. gigantica*. Worms were washed repeatedly with physiological saline (0.9% NaCl) to remove host debris. Eggs were collected from the gall bladder of infected cattle and processed for ovicidal assays [27].

### Preparation of plant extracts

**Green tea extract:** Dried green tea leaves were ground into a powder and extracted by maceration with 50% ethanol at a 1:8 (w/v) ratio. The mixture was allowed to stand with occasional stirring, filtered, and concentrated using a rotary evaporator (IKA Rotary Evaporator RV 10, IKA Works GmbH & Co. KG, Staufen, Germany) at 70°C–80°C to obtain a viscous extract [28]. The extract yield was recorded as a percentage by weight (% w/w) relative to the initial dry plant material.

**Turmeric extract:** Dried turmeric rhizomes were pulverized and macerated with 96% ethanol at a ratio of 100 g powder to 800 mL solvent for 24 h with intermittent stirring. The filtrate was separated from the residue and concentrated using the same rotary evaporator (IKA Works GmbH & Co. KG) at 70°C–80°C to obtain a thick extract [29]. The final yield (% w/w) was also documented to support reproducibility.

### Preparation of control solutions

Nitroxynil (Fluconix-340®, MSD Animal Health, Boxmeer, the Netherlands) was used as a positive control. Nitroxynil was selected instead of triclabendazole because it is a commercially established fasciolicidal agent with proven efficacy against adult *Fasciola* spp. and is widely available in the local veterinary setting, making it an appropriate comparative reference for the present study. The working solution (10%) was prepared by diluting the commercial formulation with physiological saline, in accordance with the manufacturer’s specifications and previous studies. Physiological saline (0.9% NaCl) served as the negative control [30].

### Identification of *F. gigantica*

### Preparation of F. gigantica eggs

Gall bladder contents containing *F. gigantica* eggs were diluted with tap water and homogenized. The suspension was centrifuged at 1,184 × *g* for 10 min, and the supernatant was discarded. This washing step was repeated until a clear suspension was obtained. Egg concentration was adjusted to a minimum of 1000 eggs/mL and stored at 4°C for no longer than 24 h before use in ovicidal assays [31].

### Experimental treatments

Six experimental groups were established as follows:

- P0: Physiological saline (negative control)
- P1: Nitroxynil 10% (positive control)
- P2: 0.15% green tea extract + 0.12% turmeric extract
- P3: 0.30% green tea extract + 0.12% turmeric extract
- P4: 0.15% green tea extract + 0.24% turmeric extract
- P5: 0.30% green tea extract + 0.24% turmeric extract

All concentrations represent final concentrations (w/v) in the incubation medium. The selected concentrations were based on previous literature reports and preliminary pilot observations indicating observable adulticidal and ovicidal activities without immediate non-specific precipitation or medium instability. Each treatment was performed in four replicates, determined using the Federer formula.

### Ovicidal assay

Equal volumes (1 mL) of egg suspension and treatment solution were mixed in test tubes, covered with aluminum foil, and incubated at 28°C for 14 and 16 days. The incubation temperature of 28°C was selected because it falls within the standard embryonation range reported for *Fasciola* spp. eggs (26°C–28°C). On day 14, samples were exposed to artificial light for 2 h under standardized laboratory illumination conditions to stimulate miracidial development.

Egg morphology was examined microscopically on day 16. Eggs were classified as degenerated based on eggshell damage, nuclear disintegration, or premature operculum opening. A minimum of 100 eggs per replicate was evaluated microscopically, resulting in at least 400 eggs per treatment group. Ovicidal activity was expressed as the percentage of damaged eggs [32].

### Anthelmintic assay on adult worms

Adult *F. gigantica* worms (five worms per Petri dish) were immersed in treatment solutions. Worm mortality was assessed at 5, 10, 20, 40, 80, 160, and 320 min. These time points were selected to capture both rapid and delayed mortality kinetics and to enable comparison with the rapid onset of the positive control.

Worms were considered dead when no muscular response was observed following mechanical stimulation. Mechanical stimulation was performed by gently probing the anterior and mid-body tegument using a sterile blunt dissecting needle for approximately 5 s per worm. Absence of visible contraction or movement after stimulation was recorded as mortality. Dead worms were collected for histological and ultrastructural analyses [27].

### Histopathological examination

Dead worms were fixed in 10% buffered formalin, processed using routine histological techniques, and stained with hematoxylin and eosin. At least one representative worm from each replicate was processed for histological examination. Tissue sections were examined under a light microscope to assess structural alterations in the tegument and internal organs [33].

### SEM

For SEM analysis, worm samples were fixed in 2.5% glutaraldehyde for 3 h, washed with phosphate-buffered saline (pH 7.4), post-fixed with osmium tetroxide for 1 h, dehydrated through a graded ethanol series, and dried using critical point drying. Samples were coated with carbon and examined using a SEM to evaluate surface ultrastructural changes [34]. Representative worms from each treatment group were selected for SEM evaluation.

### Statistical analysis

Percentage egg degeneration data and adult worm mortality data were analyzed using one-way analysis of variance (ANOVA), followed by Duncan’s multiple range test for multiple comparisons. Descriptive observations of egg morphology are presented to distinguish descriptive and analytical results. Statistical analyses were performed using SPSS Statistics version 26.0 (IBM Corp., Armonk, NY, USA). Histopathological and SEM findings were evaluated descriptively. Statistical significance was considered at p < 0.05.

## RESULTS

**Figure 1:**
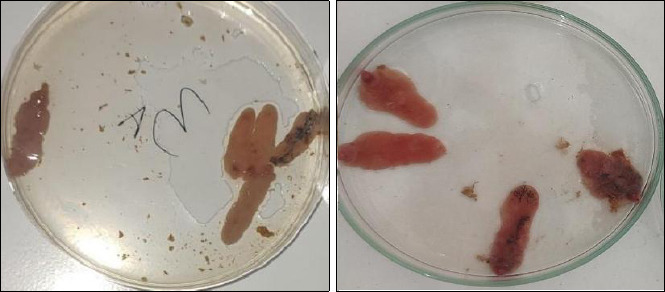
Morphological characteristics of adult *Fasciola gigantica* collected from cattle at the Ende slaughterhouse, East Nusa Tenggara, Indonesia.

Morphological examination of adult flukes collected from the gall bladders of cattle slaughtered at the Ende abattoir, East Nusa Tenggara, Indonesia, confirmed the species as *F. gigantica*. The specimens exhibited an elongated body shape, broad shoulders, and a prominent cephalic cone characteristic of *F. gigantica*, distinguishing them from *F. hepatica*, which typically has a shorter, broader morphology (Figure 1).

### Ovicidal activity of combined extracts

Microscopic evaluation of *F. gigantica* eggs after 16 days of incubation revealed distinct morphological alterations among treatments. Eggs in the negative control group (physiological NaCl) showed normal development and hatching, indicated by operculum opening and larval emergence. Similar developmental patterns were observed in the lowest extract combination (0.15% *C. sinensis* + 0.12% *C. longa*).

**Figure 2:**
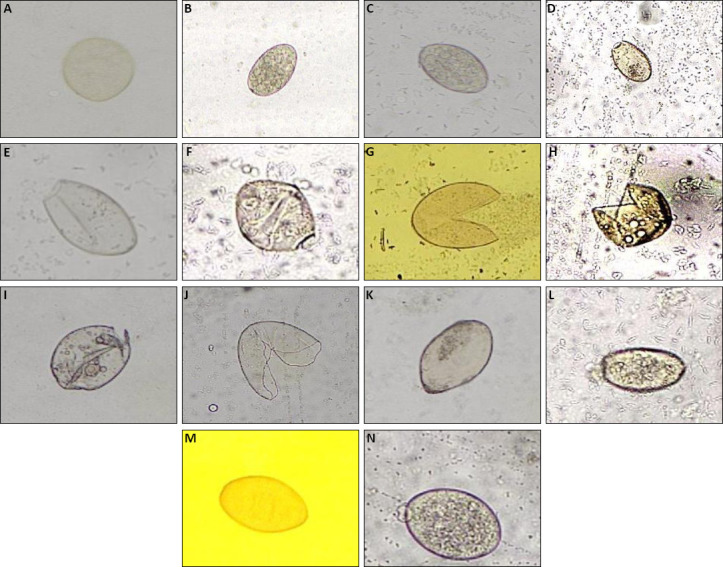
Representative micrographs of *Fasciola gigantica* eggs before and after 16 days of treatment showing normal development, hatching, membrane rupture, degeneration, and lysis (100×–400×). (A) Untreated *F. gigantica* eggs before incubation, (B and C) developing eggs after treatment, (D and E) hatching eggs, (F) eggs showing morphological deformation, (G–J) eggs exhibiting eggshell damage, (K) lysed egg, (L) undeveloped egg with opened operculum, and (M and N) undeveloped eggs without embryonic progression.

In contrast, eggs exposed to nitroxynil and higher concentrations of combined extracts exhibited severe structural damage, including eggshell rupture, membrane degeneration, lysis of egg contents, abnormal egg shape, and failure to develop. The most pronounced ovicidal effect was observed in the 0.30% *C. sinensis* + 0.24% *C. longa* treatment (Figure 2).

### Quantitative ovicidal effects

The proportion of damaged eggs increased significantly with increasing extract concentration (Tables 1 and 2). Nitroxynil induced complete egg damage (100%), whereas physiological NaCl showed the lowest damage rate.

**Table 1:** Microscopic evaluation of *F. gigantica* eggs after 16 days of incubation (%).

Treatment	Undeveloped	Developed	Hatched	Membrane damage	Degeneration
NaCl (P0)	13.24	69.03	0.93	12.52	4.28
Nitroxynil (P1)	21.64	0.00	0.00	77.36	1.00
Tea 0.15% + Turmeric 0.12% (P2)	36.09	37.12	2.65	17.02	7.12
Tea 0.30% + Turmeric 0.12% (P3)	39.93	26.39	1.45	19.62	12.60
Tea 0.15% + Turmeric 0.24% (P4)	52.71	13.58	0.00	19.97	13.74
Tea 0.30% + Turmeric 0.24% (P5)	31.14	5.97	0.00	42.94	19.95

**Table 2:** Mean percentage of egg damage after 16 days of incubation.

Treatment	Egg damage (%)
NaCl (P0)	30.04 ± 8.21ᵃ
Nitroxynil (P1)	100.00 ± 0.00ᵉ
P2	60.23 ± 10.51ᵇ
P3	72.16 ± 8.73ᶜ
P4	86.42 ± 4.01ᵈ
P5	94.03 ± 2.38ᵈᵉ

Different superscript letters indicate significant differences (p < 0.05).

At the highest extract concentration (P5: 0.30% green tea + 0.24% turmeric), the mean percentage of egg damage reached 94.03 ± 2.38%, representing the highest ovicidal activity among all extract-treated groups. Among the extract combinations, the highest egg damage was observed in the 0.30% *C. sinensis* + 0.24% *C. longa* group.

One-way ANOVA demonstrated a significant effect of treatment on egg damage (p < 0.05). Duncan’s multiple range test indicated significant differences between the control group and all extract-treated groups, as well as dose-dependent differences among extract combinations (Table 2). For clarity, Table 1 presents descriptive morphological egg outcomes, whereas Table 2 summarizes the inferential statistical analysis of total egg damage percentages.

### Anthelmintic activity against adult *F. gigantica*

Adult fluke mortality increased progressively with exposure time and extract concentration (Table 3). The positive control, nitroxynil, induced 100% mortality within the first 5 min of exposure. In contrast, the combined extracts exhibited gradual and time-dependent mortality, with increasing efficacy at later observation points.

**Table 3:** Percentage mortality of adult *Fasciola gigantica* over time.

Treatment	5 min	10 min	20 min	40 min	80 min	160 min	320 min
NaCl (P0)	0	0	0	0	0	5	20
Nitroxynil (P1)	100	100	100	100	100	100	100
P2	0	0	10	20	30	80	100
P3	0	10	20	30	30	70	100
P4	0	15	20	20	40	70	100
P5	0	15	25	40	45	90	100

Analysis of variance revealed significant effects of treatment and exposure time on mortality (p < 0.05). At 160 min, mortality induced by P2 and P5 was not significantly different from nitroxynil, whereas P3 and P4 showed numerically similar but statistically different values according to Duncan’s multiple range test.

The highest concentration (P5) produced 90% mortality at 160 min and 100% mortality at 320 min, approaching the efficacy of nitroxynil only at prolonged exposure times. No complete mortality was observed in the NaCl control.

At 160 min, mortality values in P2 (80%), P3 (70%), P4 (70%), and P5 (90%) were substantially higher than at earlier time points. Based on post hoc analysis, the lack of significant difference from nitroxynil was specifically observed in P2 and P5, whereas P3 and P4 showed numerically similar but statistically distinguishable values under the applied Duncan’s multiple range test.

This finding indicates that although the combined extracts exhibited slower onset kinetics than nitroxynil, higher concentrations achieved near-complete adulticidal activity at later time points.

### Histological alterations

**Figure 3:**
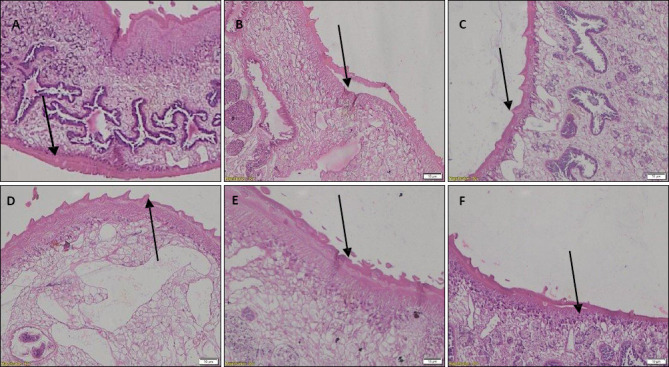
Histological alterations of tegument structure in adult *Fasciola gigantica* following treatment (hematoxylin and eosin staining, 100×–400×). (A and C) Intact tegument observed in P0 and P2, respectively, (D and E) partial tegument separation observed in P3 and P4, respectively, and (B and F) severe tegument separation observed in P1 and P5, respectively.

**Figure 4:**
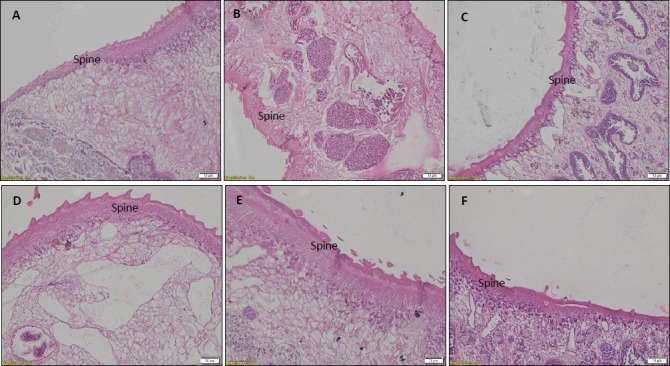
Morphological alterations of tegumental spines in adult *Fasciola gigantica* following treatment. (A, C, and D) Normal spine morphology with pointed tips observed in P0, P2, and P3, respectively, (B and E) rounded and partially detached spines observed in P1 and P4, respectively, and (F) rounded spines embedded within swollen tegument observed in P5.

**Figure 5:**
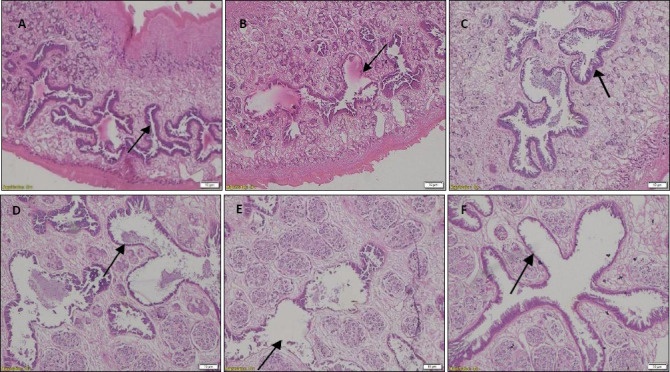
Histological appearance of the intestinal lumen in adult *Fasciola gigantica* following treatment. Arrow indicates intestinal lumen. (A and C) Normal intestinal lumen observed in P0 and P2, respectively, whereas (B, D, E, and F) ruptured intestinal lumen with villi loss observed in P1, P3, P4, and P5, respectively.

Histological examination revealed progressive structural damage correlated with extract concentration (Figures 3–5).

Control worms exhibited intact tegument, normal spines, and preserved intestinal lumen. In contrast, nitroxynil and higher extract concentrations caused tegument separation, spine deformation, and intestinal lumen rupture with villi loss.

The most severe histological damage was consistently observed in P5 and the nitroxynil-treated group, supporting the quantitative mortality findings presented in Table 3.

### Ultrastructural changes revealed by SEM

**Figure 6:**
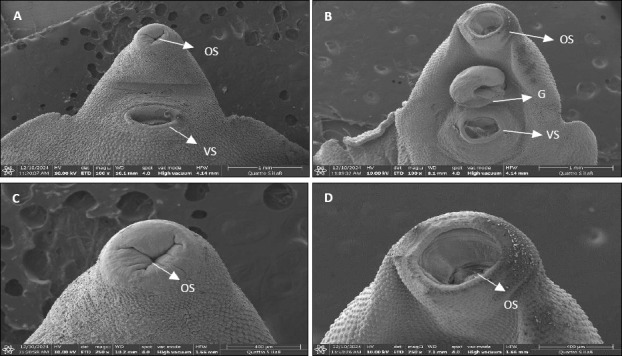
Scanning electron microscopic images of oral and ventral suckers in control and nitroxynil-treated adult *Fasciola gigantica* worms (100× magnification). (A and C) Smooth and intact oral sucker morphology observed in the negative control group (P0), whereas (B and D) widened and peeled oral sucker structures observed in the nitroxynil-treated group (P1). OS = Oral sucker, VS = Ventral sucker, G = Genital pore.

**Figure 7:**
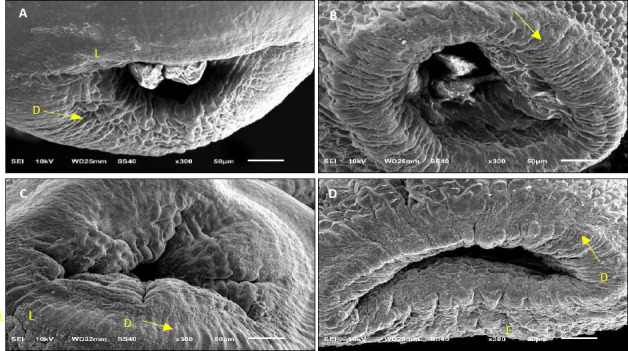
Ultrastructural alterations of oral suckers in extract-treated adult *Fasciola gigantica* worms (300× magnification). (A) P2, (B) P3, (C) P4, and (D) P5. L = Tegument peeling, D = Tegument distortion, E = Tegument erosion.

**Figure 8:**
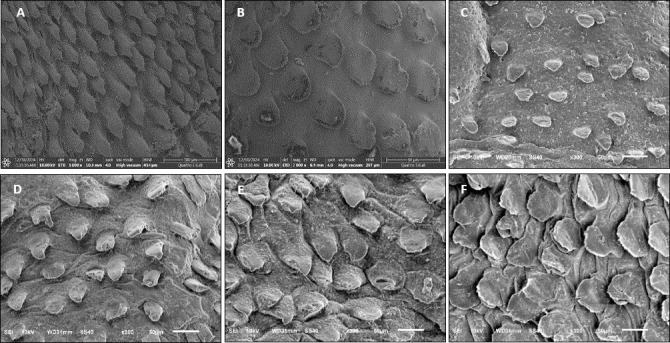
Scanning electron microscopic visualization of tegumental spine deformation in adult *Fasciola gigantica* following treatment (300× magnification). (A) Normal pointed spine morphology observed in the physiological NaCl treatment group (P0), (B) swollen spines observed in the nitroxynil-treated group (P1), and (C–F) swollen and distorted spines observed in P2, P3, P4, and P5, respectively.

**Figure 9:**
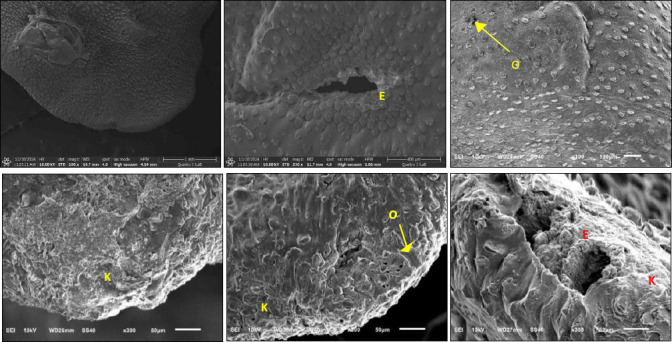
Scanning electron microscopic visualization of tegument surface damage in adult *Fasciola gigantica*, including erosion, spine loss, and exposed pores. (A) Normal tegumental surface without detectable damage observed in P0, whereas (B–F) tegumental erosion and structural damage were observed in worms treated with nitroxynil and combined green tea and turmeric extracts at different concentrations. O = Pore formed following spine detachment, K = Eroded spine, E = Tegument erosion.

SEM analysis demonstrated severe ultrastructural damage in extract-treated worms, including tegument erosion, spine distortion, oral and ventral sucker deformation, and surface exfoliation (Figures 6–9). Damage severity increased with extract concentration and closely resembled that induced by nitroxynil. Notably, P5 demonstrated marked tegumental peeling, spine swelling, and surface erosion, consistent with its higher adulticidal activity at 160–320 min.

## DISCUSSION

### Ovicidal effects of combined extracts

The present study demonstrated that the combined ethanolic extracts of green tea and turmeric exhibited significant ovicidal and anthelmintic activities against *F. gigantica* *in vitro*. Although the combined extracts showed marked biological activity, their efficacy was lower and their onset slower than that of the commercial anthelmintic nitroxynil, particularly at the early observation time points (Table 3). These effects were evidenced by marked egg damage, increased adult fluke mortality, and pronounced histological and ultrastructural alterations. Collectively, the findings support the potential of this herbal combination as a natural alternative to synthetic anthelmintics and warrant further *in vivo* investigation before practical application can be recommended.

The ovicidal activity observed in this study was characterized by eggshell rupture, membrane degeneration, abnormal morphology, and lysis of egg contents (Figure 2; Table 1). These alterations are consistent with previous studies evaluating plant-based anthelmintics against *F. gigantica* eggs, in which damage to the eggshell and embryonic degeneration were considered indicators of ovicidal efficacy [35]. The similarity of egg damage patterns between extract-treated groups and the nitroxynil control suggests that the combined extracts may act through comparable structural disruption of the egg envelope [36].

The ovicidal activity of the extracts is likely attributable to the combined, potentially additive, effects of tannins from green tea and curcuminoids from turmeric. Tannins are known to bind structural proteins in the eggshell, impair membrane integrity, alter permeability, and interfere with embryogenesis, ultimately preventing larval development [37]. Meanwhile, curcumin, a major bioactive curcuminoid in turmeric, has been reported to induce oxidative stress, membrane destabilization, mitochondrial dysfunction, and cellular degeneration in helminth eggs [38]. In addition, curcuminoids may disrupt intracellular redox balance and impair key enzymatic pathways necessary for embryonic development. The concentration-dependent increase in egg damage observed in this study (Table 2) further supports a direct chemical interaction between these phytochemicals and egg structures [39].

The 30.04% egg damage observed in the NaCl negative control (Table 2) was relatively high and may reflect the inherent variability of field-collected egg samples obtained from naturally infected abattoir-derived material. Variations in egg age, pre-existing developmental status, and minor handling-associated mechanical stress during processing may have contributed to baseline degeneration. Therefore, this finding should be interpreted as biological variability rather than treatment-related activity.

The incubation period (16 days at 28°C) was selected to coincide with the known embryonation window of *Fasciola* spp. eggs. Increased ovicidal efficacy at later observation points aligns with previous reports indicating progressive egg vulnerability during embryonic development [40]. These findings suggest that the combined extracts could potentially be applied as natural environmental disinfectants to interrupt parasite transmission in endemic areas [41].

### Anthelmintic activity against adult *F. gigantica*

Adult fluke mortality increased with both extract concentration and exposure time, indicating a time-dependent anthelmintic effect (Table 3). Although nitroxynil induced rapid mortality within 5 min (100%), the combined extracts demonstrated substantial efficacy, achieving complete mortality within 320 min at all tested concentrations [42]. Therefore, the extract activity should be interpreted as approaching or becoming comparable to nitroxynil only at prolonged exposure times rather than across all observation points.

Importantly, at later time points (160–320 min), mortality induced by higher extract combinations, particularly P5, approached the efficacy of nitroxynil, suggesting comparable efficacy under prolonged exposure [40]. This distinction is important because the kinetic profiles differed considerably between the herbal extracts and the positive control.

The delayed onset of action observed in extract-treated groups compared with nitroxynil may reflect differences in pharmacodynamic properties. Synthetic anthelmintics typically exert rapid neuromuscular paralysis, whereas plant-derived compounds may require longer exposure to disrupt metabolic, neuromuscular, oxidative, or membrane-associated processes [43]. Nevertheless, the progressive increase in mortality across extract combinations indicates that even lower concentrations possess measurable bioactivity against adult flukes [44].

The use of physiological NaCl as a negative control confirmed that the mortality observed in the treated groups was attributable to the extracts rather than to environmental stress alone. Although limited mortality was observed in the control group at later time points (20% at 320 min; Table 3), this is likely attributable to prolonged *in vitro* maintenance conditions rather than toxic effects [45].

### Histological and ultrastructural alterations

Tegumental damage is widely recognized as a critical indicator of anthelmintic efficacy in trematodes. In the present study, histological examination revealed tegument separation, spine deformation, and intestinal lumen disruption in extract-treated worms (Figures 3–5). These alterations compromise nutrient absorption, osmoregulation, and host–parasite interactions, ultimately leading to parasite death [46].

SEM analysis provided detailed confirmation of these structural changes, revealing tegument erosion, spine swelling, spine loss, and deformation of oral and ventral suckers (Figures 6–9). Such damage likely facilitates increased permeability of the tegument, allowing deeper penetration of bioactive compounds and exacerbating internal tissue damage [47]. Similar ultrastructural disruptions have been reported following exposure to both synthetic anthelmintics and plant-derived compounds, supporting the hypothesis that tegumental integrity is a primary target of anthelmintic action [48].

The observed spine distortion and loss may also impair parasite attachment and locomotion, further contributing to mortality. Previous studies have reported that spinal damage is associated with osmotic stress and disruption of the tegumental syncytium, which may explain the progressive deterioration observed as extract concentration increases [49].

### Synergistic effects and practical implications

The combined use of green tea and turmeric extracts appeared to show enhanced activity compared with several reports in the literature describing the use of individual plant extracts. However, because this study did not include single-extract treatment groups tested in parallel, a true synergistic interaction cannot be conclusively demonstrated. Therefore, the observed effect is more appropriately described as a potential additive or enhanced combined effect rather than confirmed synergy. This clarification is important in interpreting the biological interaction between the two plant extracts.

The enhanced activity may result from complementary mechanisms of action, including protein binding by tannins and oxidative, metabolic, or membrane-disrupting effects of curcuminoids [50]. Such combined mechanisms support the rationale for using multi-plant formulations in helminth control strategies [51].

A further limitation of the present study is the absence of phytochemical profiling of the final extracts. Quantitative confirmation of key bioactive constituents, such as epigallocatechin gallate and curcuminoids, would strengthen the mechanistic interpretation of the observed biological effects and improve reproducibility [52].

### Limitations and future perspectives

This study was conducted exclusively under *in vitro* conditions, which may not fully replicate the complexity of host–parasite interactions *in vivo*. Therefore, further studies are needed to evaluate *in vivo* efficacy, pharmacokinetics, safety, and optimal dosing strategies. Future studies should also include single-extract control groups, dose–response modeling, and phytochemical quantification to better define the contribution of each extract component.

Additionally, molecular investigations targeting parasite genes involved in metabolism, proteolysis (e.g., cathepsin L), and tegument maintenance would provide valuable insights into the precise mechanisms underlying the observed effects [53].

Despite these limitations, the findings highlight the promise of combined green tea and turmeric extracts as environmentally friendly, accessible, and cost-effective alternatives to synthetic anthelmintics, particularly for small-scale livestock systems in endemic regions.

## CONCLUSION

The combined ethanolic extracts of green tea and turmeric exhibited significant ovicidal and anthelmintic activities against *F. gigantica* *in vitro*. The combined extracts produced concentration- and time-dependent effects, characterized by severe egg degeneration, increased adult worm mortality, and marked histological and ultrastructural alterations involving the tegument, spines, suckers, and intestinal lumen. Among the tested treatments, the highest extract concentration (P5) exhibited the greatest biological activity, resulting in 94.03 ± 2.38% egg damage and complete adult worm mortality at 320 min. Although the efficacy of the combined extracts approached that of nitroxynil at prolonged exposure times, their onset of action remained slower than that of the commercial anthelmintic.

The findings of this study provide practical evidence that combined plant-derived extracts may serve as environmentally friendly, potentially sustainable alternatives to synthetic anthelmintics for the control of fasciolosis. The use of green tea and turmeric extracts may be particularly beneficial for livestock systems in endemic regions where anthelmintic resistance and limited access to commercial drugs are increasing concerns.

A major strength of this study was the integrated evaluation of ovicidal activity, adulticidal efficacy, histopathological alterations, and SEM-based ultrastructural damage within a single experimental framework. This comprehensive approach provided broader mechanistic insight into the antiparasitic effects of the combined extracts against multiple developmental stages of *F. gigantica*. However, the study was limited by its exclusive *in vitro* design, absence of phytochemical quantification, and lack of single-extract comparator groups, which prevented definitive confirmation of synergistic interactions between the two plant extracts.

Future studies should therefore focus on *in vivo* efficacy, safety assessment, pharmacokinetics, phytochemical characterization, dose optimization, and molecular investigations targeting parasite metabolism and tegument-associated pathways. Comparative studies involving single and combined extracts are also necessary to clarify the extent of additive or synergistic interactions.

Overall, the present findings indicate that combined green tea and turmeric extracts possess promising antiparasitic potential against *F. gigantica* and may contribute to the future development of plant-based anthelmintic strategies for sustainable fasciolosis management.

## DATA AVAILABILITY

All data generated or analyzed during this study are included in this published article. Additional supporting data may be obtained from the corresponding author upon reasonable request.

## AUTHORS’ CONTRIBUTIONS

SLH and EPH: Conceptualized and supervised the study and drafted the manuscript. LTS: Data curation, drafted the manuscript, and formal analysis. KR and DR: Investigation, visualization, and drafted the manuscript. AJ and ARK: Methodology, formal analysis, and drafted the manuscript. NH: Conceptualized the study, validation, and drafted the manuscript. MM: Investigation, data curation, and manuscript drafting, review, and editing. All authors have read, reviewed, and approved the final manuscript.
